# Shared molecular mechanisms in aging and neurodegenerative diseases: from mechanistic insights to therapeutic strategies

**DOI:** 10.3389/fnhum.2026.1768774

**Published:** 2026-05-01

**Authors:** Yesim Kaya, Kevser Kübra Kırboğa

**Affiliations:** 1 Department of Molecular Biology and Genetics, Faculty of Science, Mugla Sitki Kocman University, Muğla, Türkiye; 2 Department of Bioengineering, Faculty of Engineering, Bilecik Şeyh Edebali University, Bilecik, Türkiye

**Keywords:** brain aging, genomic instability, telomere attrition, loss of proteostasis, mitochondrial dysfunction, inflammation, neurodegenerative diseases, therapeutic strategies

## Abstract

Aging is a complex biological process characterized by progressive functional decline and increased vulnerability to age-related diseases, particularly neurodegenerative disorders. At the biological level, aging is characterized by a range of molecular and cellular mechanisms, including genomic instability, telomere attrition, loss of proteostasis, mitochondrial dysfunction, and chronic inflammation, which collectively contribute to cognitive decline and neuronal dysfunction over time. These hallmarks do not function independently but instead interact with one another during aging and neurodegeneration. Consequently, brain aging and neurodegenerative diseases are recognized as closely interconnected processes. To better understand this relationship, it is essential to examine the shared molecular and cellular mechanisms that link brain aging to neurodegeneration. In this review, we summarize the principal mechanisms underlying aging and neurodegenerative diseases, examine their roles in these processes, and highlight how their interactions shape both aging and neurodegeneration. We also discuss potential therapeutic strategies targeting key mechanisms involved in aging and neurodegeneration.

## 1 Introduction

Aging is a complex, progressive, and irreversible biological process involving numerous interrelated molecular and cellular mechanisms. During this process, cellular functions are gradually lost as a result of the progressive accumulation of cellular damage over time. The systemic deterioration of multiple tissues during biological aging is significantly associated with an increased risk of age-related diseases (Li et al., 2024; Giaimo and Traulsen, 2022; Sanada et al., 2025). Notably, the rate of aging is not uniform; it varies across species, among individuals within the same species, and even between different tissues within a single organism (Carmona and Michan, 2016). This biological heterogeneity underscores the multifactorial and multi-scale nature of the aging process, which manifests at molecular, cellular, and systemic levels and increases susceptibility to numerous pathologies, including cancer, cardiovascular diseases, and neurodegenerative conditions (Li et al., 2024; Mo, 2024).

From a demographic perspective, population aging has emerged as one of the most significant global challenges. A historical perspective suggests that, by 1950, no country had observed a percentage of the population aged 65 years and over exceeding 11% of the total population. By 2000, this rate had reached 18%. Furthermore, projections indicate that by 2050, the population aged 60 years and over will statistically outnumber the young population aged 10–24 years (Navaneetham and Arunachalam, 2025; Rudnicka et al., 2020). As reported by the World Health Organization (WHO), the number of individuals aged 60 years and older is projected to reach 2 billion by 2050. This demographic transition has led to a significant increase in the prevalence of age-related pathologies, the incidence of which approximately doubles every 5 years after the age of 60. Consequently, this trend constitutes a major public health issue on a global scale (World Health Organization, 2023; Melzer et al., 2019; Pandey, 2025). These findings indicate that maintaining healthy aging is important for promoting public health and increasing life expectancy, and current research has increasingly focused on identifying the fundamental causes of aging and developing effective and safe therapeutic interventions (Mo, 2024; Li et al., 2024).

**Figure 1:**
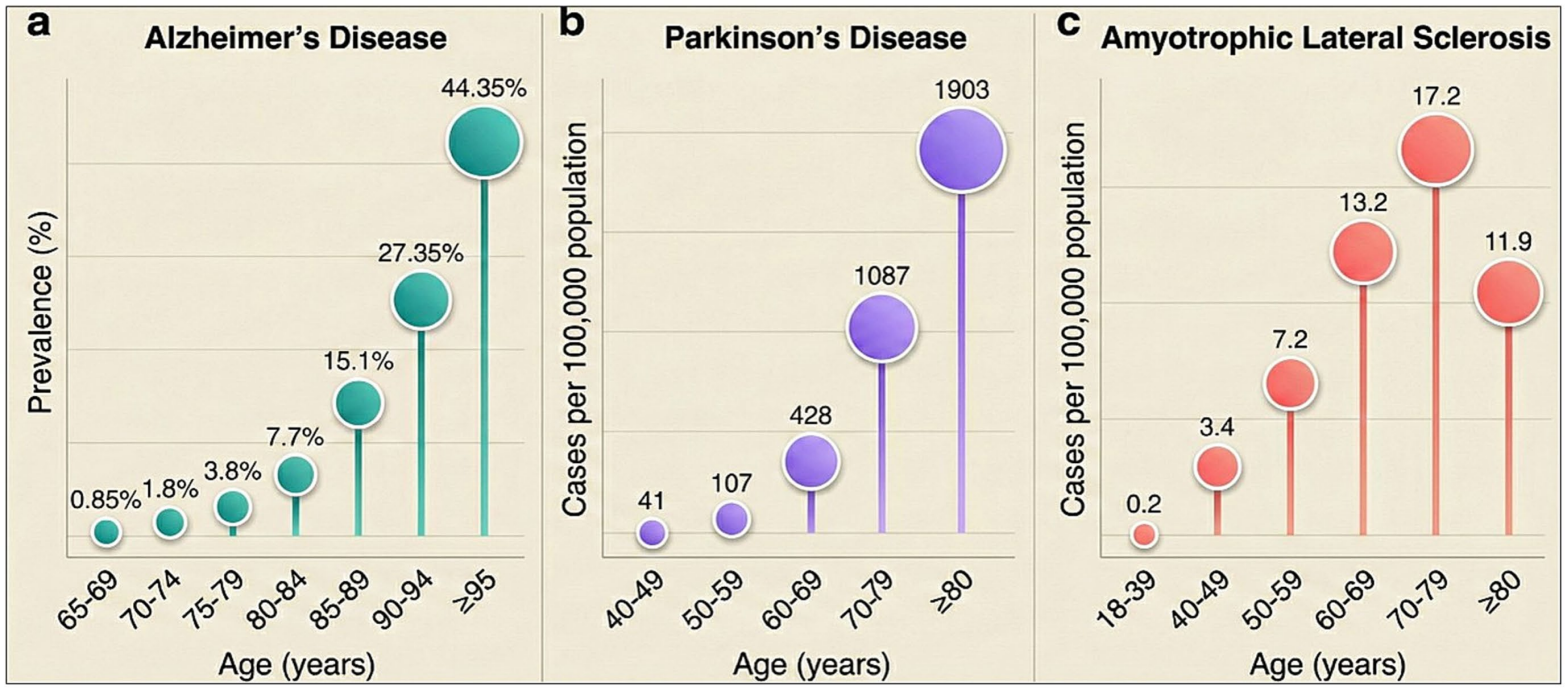
Prevalence of neurodegenerative diseases. **(a)** Alzheimer’s disease per 1,000 people in the USA for both men and women, **(b)** Parkinson’s disease per 100,000 people globally for both men and women, **(c)** ALS per 100,000 population in the USA based on 2014 data, categorized by age group.

The aging process is considered one of the primary risk factors for common neurodegenerative disorders (Zia et al., 2021). Neurodegenerative diseases, which are characterized by the degeneration of neuronal structure and loss of function, are among the major causes of disability and mortality worldwide, particularly in older individuals. These diseases impose serious social and economic burdens on patients and their families (Luhovyi and Bayliak, 2024; Kesidou et al., 2023). The increased risk of neurodegenerative diseases in older populations demonstrates a strong association between these disorders and the biological mechanisms of aging (Azam et al., 2021; Kesidou et al., 2023). Studies indicate that the prevalence of Alzheimer’s disease (AD) in the USA increases significantly with age in both male and female populations, reaching its highest levels in the oldest age groups (Figure 1a) (Alzheimer’s Association, 2018; Nussbaum and Ellis, 2003; Hy and Keller, 2000). Similarly, global findings show that the prevalence of Parkinson’s disease steadily increases with age in both sexes, with the highest incidence observed among the oldest individuals (Figure 1b) (Nussbaum and Ellis, 2003; Poewe et al., 2017). Consistent with this pattern, the prevalence of Amyotrophic lateral sclerosis (ALS) (per 100,000 population, 2014) increases with age, becomes more pronounced in middle to late adulthood, and reaches its highest levels in old age (Figure 1c) (Mehta et al., 2018).

**Figure 2:**
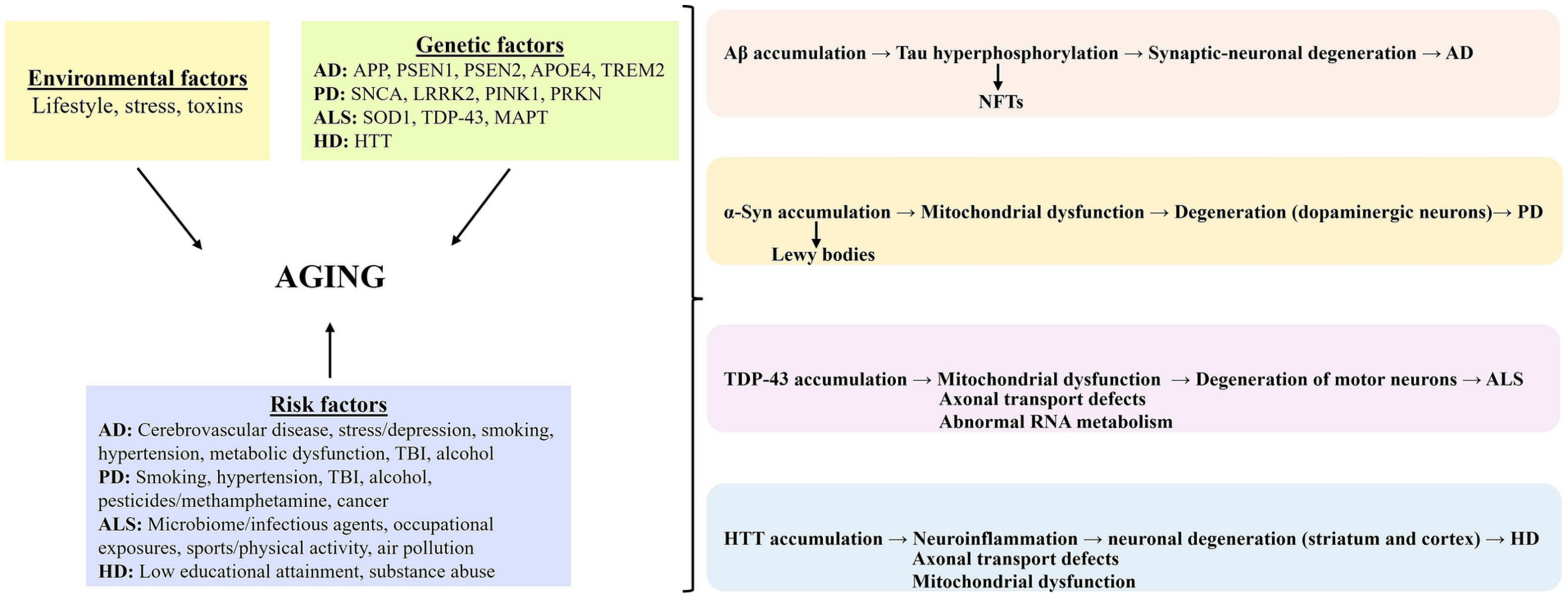
Aging, as a shared biological context, interacts with environmental, genetic, and disease-specific risk factors to drive the development of different neurodegenerative diseases.

Aging serves as the principal biological context in which environmental exposures, genetic predisposition, and disease-specific risk factors interact to facilitate the onset and progression of neurodegenerative disorders. Although aging is a broad and non-specific process, it creates a permissive background for the activation of distinct pathogenic mechanisms in different diseases (Zhou et al., 2024). In this framework, Alzheimer’s disease is characterized by Aβ accumulation, tau hyperphosphorylation, NFT formation, and synaptic-neuronal degeneration; Parkinson’s disease by *α*-synuclein accumulation, Lewy body formation, mitochondrial dysfunction, and degeneration of dopaminergic neurons; ALS by TDP-43-associated mitochondrial dysfunction, axonal transport defects, abnormal RNA metabolism, and motor neuron loss; and Huntington’s disease by HTT accumulation, neuroinflammation, impaired axonal transport, mitochondrial dysfunction, and degeneration in the striatum and cortex (Zhang et al., 2025; Leak et al., 2024; Balendra et al., 2025; Joshi et al., 2025). Thus, while aging represents a shared background condition, its interaction with specific risk profiles and molecular cascades determines the heterogeneity of neurodegenerative disease phenotypes (Figure 2) (Jiang et al., 2025) 0.50.

Cognitive abilities decline with physiological aging, but this decline does not generally impair daily functioning or normal brain function. However, AD and other neurodegenerative diseases are, in most cases, characterized by rapid cognitive decline (Jakob-Roetne and Jacobsen, 2009). Although age is considered one of the main risk factors for neurodegenerative diseases, these disorders differ from physiological aging in that they are characterized by significant losses in synaptic connections, neuron number, and neuronal function in different brain regions (Pini et al., 2016; Daniele et al., 2018). The rarity of pathology-free brains at older ages and the increased prevalence of neurodegenerative disorders suggest that brain aging may form a continuum with neurodegeneration. This observation indicates that neurodegenerative diseases may be considered a manifestation of accelerated aging (Hou et al., 2019).

To systematically understand this relationship, it is useful to consider the molecular and cellular hallmarks of aging. In a 2013 study, López-Otín and colleagues proposed nine key hallmarks of aging that interact with one another and contribute to the aging process. These hallmarks are not randomly arranged; rather, they are logically classified into three groups according to a “cause–response–effect” framework (López-Otín et al., 2013). The primary determinants of aging, including genomic instability, telomere attrition, epigenetic alterations, and loss of proteostasis, represent the fundamental causes of cellular damage and directly trigger aging. These determinants exert harmful effects at the genome, proteome, and organelle levels and contribute significantly to age-related diseases such as Werner syndrome, AD, Parkinson’s disease (PD), and Huntington’s disease (HD). Secondary (antagonistic) determinants, including cellular senescence and dysregulated metabolism, arise in response to the damage caused by primary determinants. At low intensity, these determinants may exert protective effects; however, when chronically activated, they become harmful. In conditions such as PD and AD, oxidative stress caused by mitochondrial dysfunction is a major factor. Tertiary (integrative) determinants emerge when the damage caused by primary and secondary determinants exceeds the capacity to maintain homeostasis. These determinants, including stem cell exhaustion and altered intercellular communication, contribute to changes in tissue homeostasis associated with aging and to the development of diseases such as sarcopenia, immunosenescence, and AD (López-Otín et al., 2023; Sanada et al., 2025; Tartiere et al., 2024; Tenchov et al., 2024). Although these three groups of determinants are not entirely distinct from one another, they are hierarchically related: primary determinants initiate the aging process through the accumulation of damage; antagonistic determinants may initially have beneficial effects but eventually become harmful; and integrative determinants emerge when the damage exceeds the capacity to preserve tissue homeostasis (López-Otín et al., 2013).

Therefore, elucidating the molecular mechanisms of brain aging is critically important for the development of strategies to slow the aging process and to prevent or treat age-related neurodegenerative disorders. In this review, we summarize the principal mechanisms underlying aging and neurodegenerative diseases, examine their roles in these processes, and highlight how their interactions shape both aging and neurodegeneration. We also discuss potential therapeutic strategies targeting key mechanisms involved in aging and neurodegeneration.

## 2 Shared mechanisms of aging and neurodegenerative diseases

**Figure 3:**
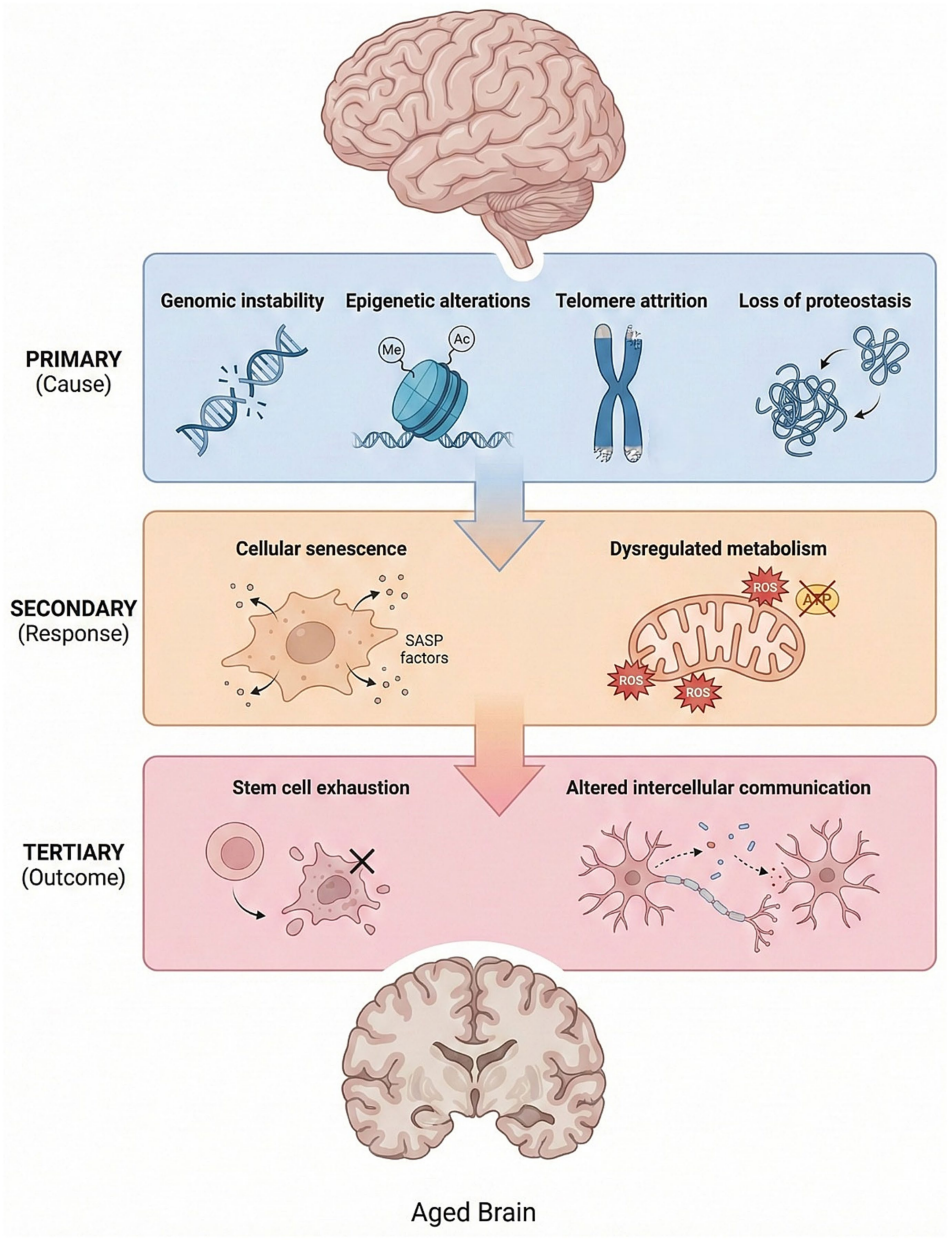
Hallmarks of brain aging. The scheme illustrates the principal hallmarks of brain aging, organized into three levels: primary, secondary, and tertiary hallmarks. Primary hallmarks include genomic instability, telomere attrition, epigenetic alterations, and loss of proteostasis. Secondary hallmarks include dysregulated metabolism and cellular senescence, which develop in response to primary damage. Tertiary hallmarks, such as stem cell exhaustion and altered intercellular communication, reflect the integrated consequences of these processes, ultimately leading to loss of tissue homeostasis and age-related brain dysfunction.

Although aging and neurodegenerative diseases have long been considered distinct processes, current evidence suggests that they share numerous molecular mechanisms. This section will summarize how the primary processes known as the “hallmarks of aging” contribute to the pathogenesis of neurodegenerative diseases. Deregulated nutrient sensing, genomic instability, epigenetic alterations, telomere attrition, loss of proteostasis, mitochondrial dysfunction, cellular senescence, stem cell exhaustion, and altered intercellular communication have emerged as common nodal points in both the progression of physiological aging and the onset and progression of neurodegenerative diseases (Figure 3). In the following subsections, these interconnected common mechanisms between brain aging and neurodegenerative diseases will be addressed in primary, secondary, and tertiary contexts.

### 2.1 Genomic instability

The genomic integrity of cells is crucial for maintaining cellular stability and ensuring healthy cellular functions. Genomic DNA is damaged by exogenous and endogenous factors, including UV radiation, oxidative stress, reactive oxygen species (ROS) and replication errors. Each cell experiences thousands of DNA lesions per day. Such DNA damage accumulates throughout an individual’s lifetime (Jackson and Bartek, 2009; Chatterjee and Walker, 2017; Hoeijmakers, 2009). Cells have developed complex DNA repair mechanisms to repair and reduce DNA damage (Lord and Ashworth, 2012). Unrepaired DNA lesions and persistent DNA damage can lead to genomic instability which is widely recognized as a fundamental hallmark of aging and represents one of the earliest molecular processes linking cellular aging to neurodegenerative disease vulnerability (López-Otín et al., 2013; Vijg and Suh, 2013).

The post-mitotic nature of neurons and their high metabolic demands make the nervous system particularly susceptible to genetic instability (Madabhushi et al., 2014). Post-mitotic neurons are highly vulnerable to DNA damage due to their long lifespan, high energy requirements, and elevated transcriptional activity (Gaspar-Silva et al., 2023). Aging neurons demonstrate increased sensitivity to ROS-mediated DNA damage, particularly as a result of excitatory synaptic activity (Jin and Cai, 2023). Furthermore, it has been demonstrated that glial cells exhibit severe pathological levels of DNA damage during aging (Vaidya et al., 2014; Pao et al., 2020).

Base excision repair (BER) is the primary mechanism for repairing oxidative DNA damage and thereby maintaining genomic integrity in post-mitotic neurons. In this context, if DNA damage is not fully repaired or exceeds the repair capacity of BER, the pathway may promote the accumulation of toxic intermediates, such as single strand DNA breaks (Frosina, 2000). During aging, BER initiation is preserved or even enhanced in specific brain regions, such as the substantia nigra, through the upregulation of certain DNA glycosylases. In contrast, the expression and activity of DNA polymerase *β* (Polβ), which is essential for BER completion, declines with age, impairing overall repair capacity (Sykora et al., 2015; Cabelof et al., 2002; Rao et al., 2000). Overall, this age-related imbalance in BER may increase neuronal genomic stress and contribute to neurodegenerative processes.

In neurodegenerative diseases, DNA damage and the ensuing genomic instability may accelerate disease progression. Genomic DNA integrity has been reported to be compromised in AD brain, and such damage may contribute to neuronal loss (Adamec et al., 1999). Single-cell studies of AD brain samples have shown that DNA damage increases with disease progression, alongside a decline in BER-mediated repair activity (Dileep et al., 2023; Weissman et al., 2009). Consistent with this, Polβ activity is reduced in the early stages of the AD and decreases further in advanced stages (Sykora et al., 2013). Hou et al. (2018) demonstrated that impaired DNA repair in 3xTg-AD mice with partial Polβ deficiency increased genomic instability by enhancing DNA damage and DNA damage response (DDR) in the brain. In the same model, this situation accelerated the progression of Alzheimer’s-like phenotypes by p-tau pathology, synaptic dysfunction, neuronal death, and cognitive impairment (Hou et al., 2018). Sykora et al. (2015) reported that reduced Polβ expression (Polβ+/−) in 3xTg-AD mice increases the accumulation of DNA damage and exacerbates neurodegenerative phenotypes. The study also showed that Polβ insufficiency is accompanied by impairments in bioenergetic and mitochondrial pathways. These findings collectively suggest that a self-reinforcing feedback loop linking oxidative stress (ROS), DNA damage, and mitochondrial dysfunction may contribute to disease progression (Sykora et al., 2015).

In the pathogenesis of PD, it is evident that the age-related accumulation of oxidative DNA lesions and the decline in DNA repair capacity with aging contribute to genomic instability. SenGupta et al. (2021), demonstrated in a *C. elegans*-based sporadic PD model that NTH-1-initiated base excision repair can increase the accumulation of repair intermediates (particularly ssDNA breaks) in mitochondrial and nuclear DNA with age. As a result, this accumulation may directly contribute to dopaminergic neuron degeneration.

It has been suggested that Aβ aggregation is one of the key pathological hallmarks of AD, can exacerbate DNA damage by increasing aberrant synaptic activity and elevating ROS production (Merlo et al., 2016). Similarly, increased oxidative DNA damage has been reported in neurons in ALS and in mitochondrial DNA in PD (Bender et al., 2006; Fitzmaurice et al., 1996). Within a broader mechanistic framework, persistent DNA damage may trigger intracellular signaling cascades in addition to genomic instability, negatively affecting sirtuins, DNA repair, mitophagy, and mitochondrial health through PARP1 activation, increased PARylation, and NAD^+^ consumption. It is further postulated that this may exacerbate age-related neurodegeneration by increasing cellular senescence and inflammation (Madabhushi et al., 2014).

Based on this information, it seems that genomic instability contributes to both aging and neurodegenerative diseases. Furthermore, it appears to interact with other hallmarks, such as Aβ aggregation, mitochondrial dysfunction, and neuroinflammation.

### 2.2 Telomer attrition

Telomeres are complexes of DNA and proteins that help to maintain genomic stability by protecting the ends of chromosomes from degradation (Shay and Wright, 2019). Telomeres are composed of tandem DNA repeats (TTAGGG), which are shielded by telomere-associated proteins throughout interphase (Zia et al., 2021; Zhang et al., 2016). This protective structure contributes to the maintenance of genomic integrity by preventing replication-associated factors, including end-to-end fusions and aberrant recombination intermediates (Muraki et al., 2012; Zia et al., 2021).

Telomere attrition is another major hallmark of aging, representing the progressive loss of telomere reserves in mitotic cells (López-Otín et al., 2013; Rossiello et al., 2022). Telomere shortening leads to activation of the DDR induced cell-cycle arrest and cellular senescence (Bernadotte et al., 2016; Rossiello et al., 2022). Telomeres progressively shorten due to insufficient telomerase activity which may accelerate aging and influence the pathogenesis of neurodegeneration. As a result of telomere attrition, a high amount of DNA damage may increase neuronal vulnerability to oxidative stress and proteotoxicity (Jinesh et al., 2025; von Zglinicki et al., 1995).

In this context, telomerase is a critical ribonucleoprotein enzyme that maintains telomere length, becoming particularly relevant. It consists of TERT and TER subunits and can support the synthesis of telomeric repeats using its own RNA template. Telomerase activity can compensate for telomere loss during cell division (Nandakumar and Cech, 2013; Jiang et al., 2018).

Elevated telomerase activity has been observed in neural stem and progenitor cells in the developing brain and, similarly, in hippocampal neurogenesis. High levels of telomerase activity are crucial for cell proliferation, neuronal survival, and differentiation (Cai et al., 2002; Liu et al., 2018).

Demanelis et al. (2020) utilized the v8 data from the Genotype-Tissue Expression (GTEx) project in their studies. In this context, telomere length was measured as relative telomere length (RTL) in a total of 6,391 post-mortem samples from more than 25 tissue types obtained from 952 donors aged 20–70. The analyses showed that telomere length (TL) was inversely related to age in most tissues, supporting the finding of widespread age-related telomere shortening in human tissues. It was also reported that the severity/rate of telomere shortening could vary between tissues, and that the age–telomere relationship was more pronounced in tissues with shorter average telomere length. Furthermore, it has been noted that TL measurements in different tissues generally show a positive correlation and that TL measured from whole blood can be used as a representative proxy for many tissues (Demanelis et al., 2020).

A number of studies with findings about telomere attrition in neurodegenerative illnesses vary by cell type and different brain regions (Table 1). Forero et al. (2016) conducted a meta-analysis of 13 case–control studies and compared telomere length in a total of 860 Alzheimer’s patients (aged 65–84) and 2,022 controls. The results showed that telomeres in AD were significantly shorter than in controls (Forero et al., 2016). In a study using an APP/PS1 mouse model exhibiting Alzheimer-like pathology, it was observed that telomeres shortened in disease-related microglia, leading to replicative senescence (Hu et al., 2021). Another study reported telomere shortening in microglia with aging and a tendency for shorter microglial telomeres in human post-mortem samples in the presence of dementia.

**Table 1:** Studies on telomere length in Alzheimer’s and Parkinson’s diseases.

**Sample**	**TL (AD/PD vs. controls)**	**Study**
Cerebellar neurons	Unchanged (AD)	Lukens et al. (2009)
Peripheral blood leukocytes	Shorter (AD)	
Leukocytes and buccal cells	Shorter (AD)	Thomas et al. (2008)
Hippocampal cells	Longer (AD)	
PBMCs	Longer (PD)	Hudson et al. (2011)
PBMCs	Longer (PD)	
Substantia nigra	Unchanged (PD)	
Hippocampal neurons	Shorter (AD)	Franco et al. (2006)
PBMCs	Unchanged (AD)	Tedone et al. (2015)
Leukocytes	Unchanged (AD)	Guan et al. (2013)
Leukocytes	Longer (PD)	Schürks et al. (2014)
Leukocytes	Shorter (PD)	Maeda et al. (2012)
Leukocytes	Unchanged (PD)	Watfa et al. (2011)
Buccal cells	Shorter (PD)	Kolyada et al. (2016)

TL, telomere length; PBMCs, peripheral blood mononuclear cells; AD, Alzheimer’s disease; PD, Parkinson’s disease.

According to Lukens et al. (2009) peripheral blood leukocytes (PBL) from older AD patients have shorter telomeres. The same study also showed that cerebellar telomere lengths were not significantly different in AD patients (Lukens et al., 2009). As demonstrated by Thomas et al. (2008), shorter telomere lengths were reported in leukocytes and buccal cells, while hippocampal cells had longer telomere structures in Alzheimer’s patients compared to controls (Thomas et al., 2008). In contrast, a study conducted with Parkinson’s patients found longer telomeres in peripheral blood mononuclear cells (PBMCs), while no change in telomere length was observed in the substantia nigra region (Hudson et al., 2011).

**Figure 4:**
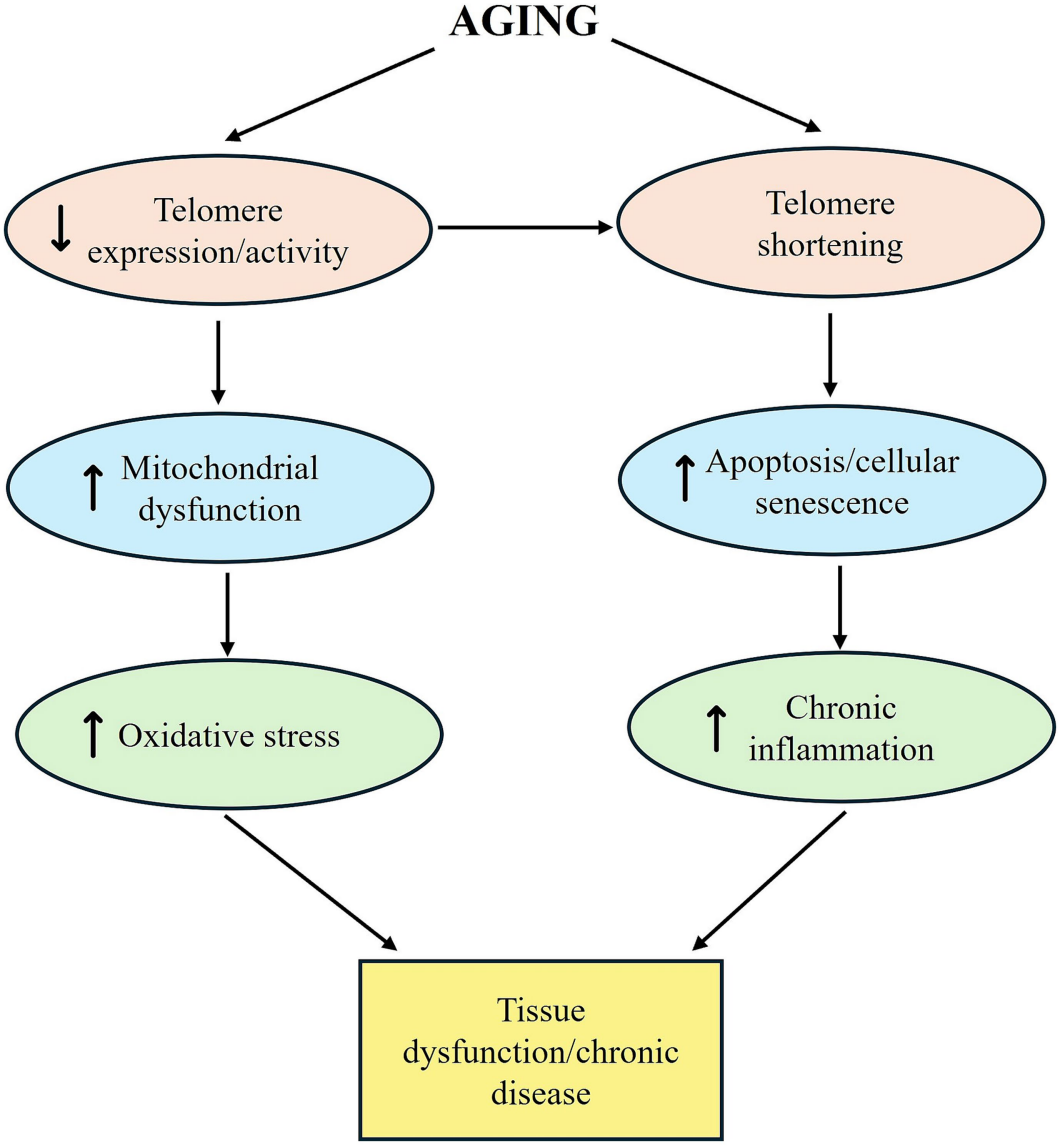
The role of telomere shortening in aging and its interactions with mitochondrial dysfunction, oxidative stress, apoptosis/senescence, chronic inflammation, and tissue dysfunction.

As with other hallmarks of aging, telomere shortening interacts with different hallmarks such as mitochondrial dysfunction, chronic inflammation, cellular senescence (Figure 4) (Zuo et al., 2019). It has been reported that telomere shortening associated with aging results in a decrease in the proliferation capacity of microglia and affects inflammatory signal transduction. In aged mice with telomere dysfunction, senescence has been reported to cause abnormal release of proinflammatory cytokines (Ju et al., 2007). Furthermore, a study using aged telomerase knockout mice suggested that telomere shortening may slow the progression of amyloid plaques in the environment and that this may be related to telomere-dependent effects on microglial activation (Rolyan et al., 2011).

### 2.3 Loss of proteostasis

Protein homeostasis (proteostasis) is critical for maintaining cellular functions. Proteostasis refers to a dynamic equilibrium that includes various processes such as protein synthesis, proper folding, post-translational modifications, intracellular transport, and the degradation of damaged or misfolded proteins. Disruption of proteostasis leads to the accumulation of misfolded and damaged proteins, which causes cellular stress and may contribute to aging and the development of neurodegenerative diseases (Mishra et al., 2025). To eliminate misfolded proteins, cells have developed protein quality control mechanisms, including molecular chaperones, the ubiquitin–proteasome system (UPS), and the autophagy–lysosomal pathway (ALP). Molecular chaperones facilitate the refolding of misfolded proteins or target them for degradation, whereas the UPS is primarily responsible for degrading short-lived, soluble, and damaged proteins. In contrast, the ALP plays a key role in the clearance of large protein aggregates, damaged organelles, and membrane-associated structures (Hipp et al., 2019; Guo et al., 2022; Ajmal, 2023; Menzies et al., 2015). Maintaining proteostasis is particularly important in non-dividing and long-lived cells. Therefore, disruption of proteostasis in neuronal cells is closely associated with neurodegenerative diseases, including Alzheimer’s disease, Parkinson’s disease, and amyotrophic lateral sclerosis (Höhn et al., 2020).

Loss of proteostasis is one of the fundamental characteristics of brain aging. With aging, the efficiency of protein quality control systems, such as molecular chaperones, the UPS, and autophagy, decreases; as a result, misfolded and damaged proteins begin to accumulate within the cell. Furthermore, increased oxidative stress and ROS-mediated damage during aging further impair the efficiency of these systems (Kasar et al., 2025; López-Otín et al., 2013). Post-mitotic neurons are particularly vulnerable to protein accumulation because they cannot reduce the burden of damaged components through cell division. As a result, aggregation-prone proteins such as tau, amyloid beta, and *α*-synuclein can accumulate in neurons. These aggregates may disrupt membrane integrity, impair intracellular transport, and contribute to neuronal death by causing synaptic dysfunction (Barmaki et al., 2023).

As in aging, impairment of autophagy and other protein quality control mechanisms in neurodegenerative diseases promotes pathological protein accumulation and neuronal dysfunction. Nilsson et al. (2013) investigated the role of autophagy in Aβ pathology using APP23 transgenic mice with conditional deletion of Atg7 in forebrain neurons. The study showed that autophagy deficiency reduced extracellular Aβ secretion and plaque formation but caused abnormal intraneuronal Aβ accumulation. Moreover, neurodegenerative changes associated with autophagy deficiency were found to worsen memory impairment in the presence of amyloid pathology (Nilsson et al., 2013). Similarly, Feng et al. (2020) demonstrated that Alzheimer’s-like tau accumulation suppresses autophagic flux, particularly through impaired autophagosome–lysosome fusion. Mechanistically, tau accumulation was reported to reduce IST1 expression, disrupt ESCRT-III complex formation, and thereby further impair tau clearance. Together, these findings suggest that a vicious cycle exists between tau accumulation and autophagy deficiency in Alzheimer’s disease, thereby exacerbating synaptic and behavioral impairments (Feng et al., 2020). In PD, the clearance of *α*-synuclein through autophagic pathways is essential for maintaining neuronal proteostasis. Vogiatzi et al. (2008) showed that wild-type α-synuclein is degraded in neuronal cells through both chaperone-mediated autophagy and macroautophagy. These findings indicate that impaired autophagy may contribute to α-synuclein accumulation and thereby to the pathogenesis of PD (Vogiatzi et al., 2008).

Loss of proteostasis is associated not only with the functional impairment of protein degradation systems, but also with mutations in the genes encoding components of these systems. Mutations in several genes encoding UPS and ALP components have been identified in neurodegenerative diseases. For example, mutations in the PRKN gene, which are associated with autosomal recessive Parkinson’s disease, can impair the function of Parkin, an E3 ubiquitin ligase involved in regulating proteins associated with cellular processes such as synaptic function and mitophagy (Shimura et al., 2000). Similarly, Hara et al. (2006) demonstrated that suppression of basal autophagy in Atg5 knockout mice leads to progressive neurodegenerative changes. These mice developed motor dysfunction, accumulated ubiquitin-positive protein aggregates and inclusion bodies in neural cells and exhibited loss of specific neuronal populations. Together, these findings indicate that Atg5-mediated autophagy plays a critical role in maintaining neuronal homeostasis (Hara et al., 2006). Beclin-1, a key regulator of autophagy, has also been implicated in the pathogenesis of both Alzheimer’s and Parkinson’s diseases. In Alzheimer’s disease, decreased Beclin-1 expression has been reported to be associated with impaired autophagic flux, particularly in the early stages of the disease (Pickford et al., 2008). In Parkinson’s disease, Beclin-1 has been shown to facilitate the clearance of toxic α-synuclein aggregates; in experimental models, upregulation of Beclin-1 reduced α-synuclein accumulation and simultaneously alleviated synaptic and dendritic damage (Spencer et al., 2009).

In conclusion, current evidence indicates that autophagy is not merely a degradative pathway for intracellular proteins and organelles, but an integral component of the proteostasis network that functionally interacts with the UPS, ER stress signaling, the unfolded protein response (UPR), immune pathways, and mitochondrial quality control. In animal models of Alzheimer’s disease, activation of autophagy has been shown to reduce neuroinflammation, improve cognitive function, enhance mitochondrial function, and alleviate oxidative stress, suggesting that loss of proteostasis is not limited to abnormal protein accumulation but is also closely linked to other aging-related processes, including immune responses, cellular stress mechanisms, and mitochondrial homeostasis (Lee et al., 2012; Hyttinen et al., 2021). Therefore, maintaining the functional balance among molecular chaperones, the UPS, and the autophagy–lysosomal pathway provides an important framework for understanding the pathogenesis of neurodegenerative diseases, while these systems also represent promising targets for therapeutic intervention.

### 2.4 Mitochondrial dysfunction and inflammation

**Figure 5:**
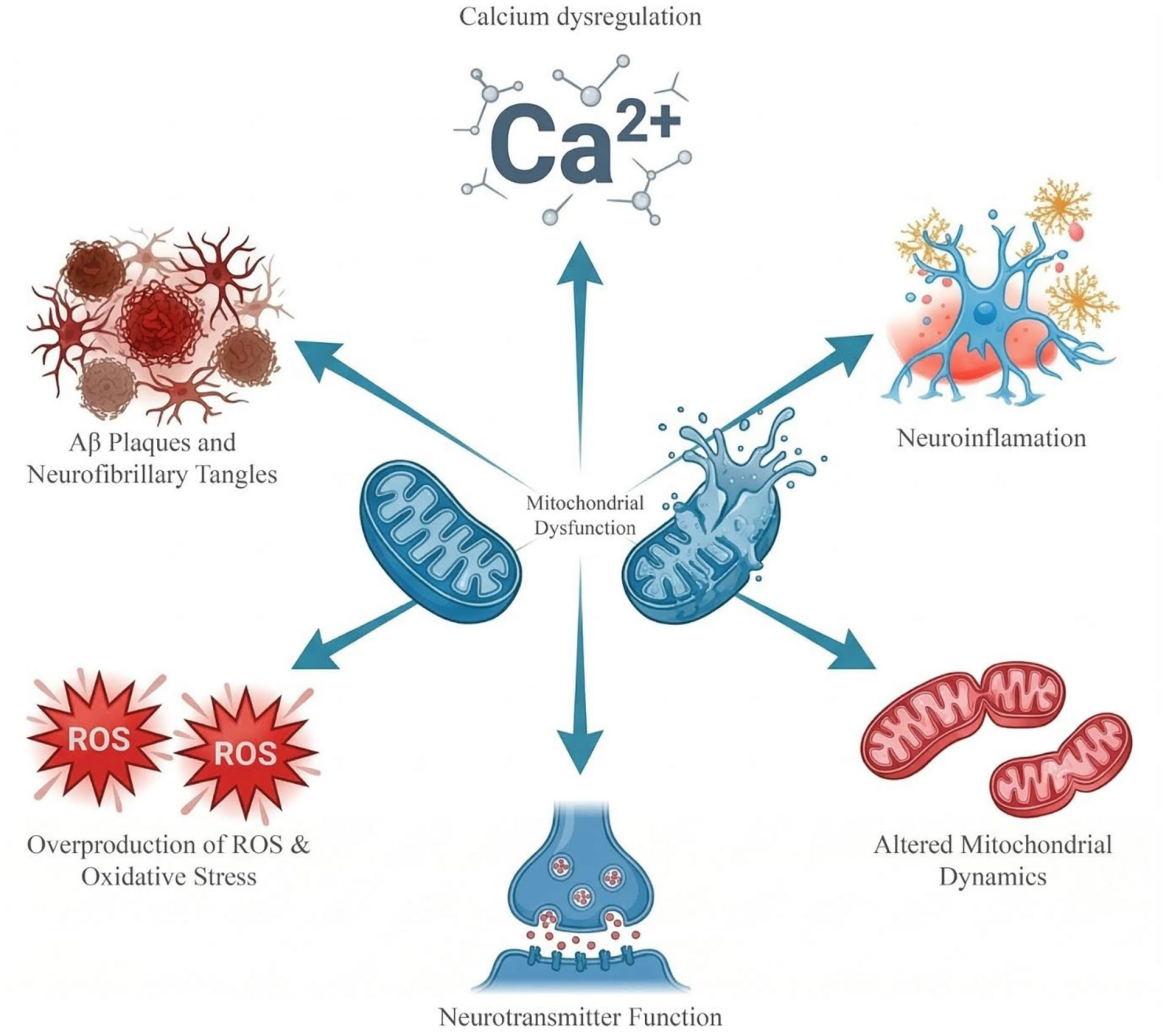
Mitochondrial dysfunction as a hallmark of aging: interactions with major neurodegenerative mechanisms.

Mitochondrial dysfunction is one of the most widely studied mechanisms underlying aging and neurodegenerative diseases. Mitochondria play a key role in cellular energy metabolism by producing ATP and the primary intracellular source of ROS generated during oxidative phosphorylation (Waltz and Giegé, 2020; Guo et al., 2023). During aging, the efficiency of mitochondrial energy production declines, leading to mitochondrial dysfunction and, consequently, increased oxidative stress. Excessive ROS production associated with mitochondrial dysfunction exceeds the capacity of antioxidant defense systems, causing damage to lipids, proteins, and DNA, particularly in post-mitotic neurons (Ullah et al., 2021; Olufunmilayo et al., 2023). This process affects not only nuclear DNA but also mitochondrial DNA (mtDNA). The accumulation of mitochondrial DNA damage further exacerbates mitochondrial dysfunction, creating a vicious cycle (Tran and Reddy, 2021). Furthermore, mitochondria become more vulnerable to structural changes during aging (Fakouri et al., 2019). Consistent with this, studies have shown that defected and dysfunctional mitochondria have been detected in aged tissues from *C. elegans*, rats, mice, and humans (Son et al., 2019; García et al., 2013; Kauppila et al., 2017). Damaged mitochondria are primarily removed through mitophagy, a process that targets them for lysosomal degradation. In addition, some mitochondrial components may also be degraded by the UPS (Saito and Sadoshima, 2015). Disruption of the balance between mitochondrial production and the clearance of damaged mitochondria may contribute to aging and neurodegenerative disorders (Cai and Jeong, 2020; Onishi et al., 2021) (Figure 5).

Disruption of intracellular calcium homeostasis plays an important role in the relationship between mitochondrial dysfunction and oxidative damage. Elevated calcium levels may lead to mitochondrial dysfunction, resulting in increased intracellular ROS production (Krieger and Duchen, 2002). Similarly, motor neurons of transgenic SOD1 G93A mice are sensitive to glutamate toxicity, resulting in oxidative DNA damage, increased intracellular calcium levels, and mitochondrial dysfunction associated with ROS production.

Glutamate toxicity can cause oxidative DNA damage, elevated intracellular calcium levels, and mitochondrial dysfunction linked to ROS generation in the motor neurons of transgenic SOD1 G93A mice. In a similar manner, glutamate toxicity can cause oxidative DNA damage, elevated intracellular calcium levels, and mitochondrial dysfunction linked to ROS generation in the motor neurons of transgenic SOD1 G93A mice (Kruman et al., 1999). Overall, these findings suggest that calcium imbalance exacerbates mitochondrial damage by increasing ROS production, which in turn creates a pathological cycle that fuels DNA damage and neuronal injury (Konopka and Atkin, 2022) (Figure 5).

Altered mitochondrial dynamics in aging and neurodegenerative diseases are associated with a disruption in the balance between fission and fusion (Rana et al., 2017; Yu et al., 2019). Impairing of this imbalance can lead to changes in mitochondrial network structure and function (Huang et al., 2007). It is shown that elongated mitochondria are observed in aging cells, while decreased p-DRP1 and increased MFN2 in aging mesenchymal stem cells suggest a shift toward fusion (Ren et al., 2020; Hong et al., 2020). In contrast, increased DRP1 and decreased OPA1 levels in aged mouse brains suggest that these changes may vary depending on the tissue (Eckert et al., 2013). Therefore, maintaining the balance between mitochondrial division and fusion is critically important for sustaining mitochondrial health and limiting age-related changes (Figure 5).

There is a strong relationship between mitochondrial dysfunction and inflammation, both in aging and in neurodegenerative pathologies. Microglia are considered the immune cells of the central nervous system. They lose their function in maintaining cellular homeostasis and shift toward a proinflammatory phenotype. As a result, the release of cytokines such as TNF-*α* and IL-1β increases, and neuronal stress increases. This chronic low-grade inflammation contributes to synaptic loss and accelerates the pathological processes seen in Alzheimer’s and Parkinson’s diseases. Moreover, astrocytic dysfunction exacerbates the neuroinflammatory environment by impairing neurotransmitter recycling and metabolic support (Gao et al., 2023; Müller and Di Benedetto, 2025). Particularly in Parkinson’s disease, it has been shown that misfolded α-synuclein triggers both mitochondrial and genomic DNA damage in microglia, followed by activation of the cGAS–STING pathway, one of the key regulators of inflammation (Hinkle et al., 2022). Furthermore, genotoxic damage accumulation and STING activation in microglia after the intrastriatal injection of misfolded *α*-synuclein in mice highlight the importance of this mechanism in the pathogenesis of PD (Standaert and Childers, 2022). Oxidative DNA damage has been reported to accumulate in both nuclear DNA and mtDNA of dopaminergic neurons in the substantia nigra in PD. Specifically, 8-oxodG levels and the accumulation of the mutant α-synuclein protein S42Y are significantly increased in the midbrain of affected individuals compared with controls (Basu et al., 2023; Gonzalez-Hunt and Sanders, 2021). When all these findings are considered together, mitochondrial dysfunction is not limited to impaired energy metabolism but also represents a fundamental mechanism driving inflammatory responses and neuroinflammation in aging and neurodegenerative diseases (Saliev, 2025) (Figure 5).

Mitochondria also play an important role in regulating intracellular calcium levels, which are involved in the control of many biological processes, particularly neuronal excitability (Bartman et al., 2024). Brown and colleagues investigated the effect of aging on the response to calcium loading of mitochondria isolated from different brain regions of aged rats. According to the results, it was found that increased Ca^2+^ levels in cortical mitochondria lead to increased ROS production and cause mitochondrial swelling. These findings suggest that aging is associated with impaired calcium buffering capacity in mitochondria and increased susceptibility to mitochondrial permeability transition pore opening. In contrast, these findings suggest that age-related mitochondrial vulnerability exhibits brain region-specific differences. Thus, this study suggests that the interaction between mitochondrial dysfunction, calcium imbalance, and aging is an important mechanism contributing to the development of neurodegenerative diseases (Brown et al., 2004) (Figure 5).

Taken together, these findings indicate that the molecular mechanisms underlying aging and neurodegenerative diseases do not act independently but rather interact with one another and collectively contribute to the onset and progression of these processes.

## 3 Mechanistic overlap in aging and neurodegeneration

The mechanisms underlying aging and neurodegenerative diseases are closely interconnected and reciprocally influence one another (Vaiasicca et al., 2024). Genomic instability, epigenetic alterations, loss of proteostasis, mitochondrial dysfunction, cellular senescence, stem cell exhaustion, and altered intercellular communication that arise during aging not only contribute to the decline of physiological functions but also promote a biological environment that supports the development of neurodegenerative diseases (Zhao and Huai, 2023). In this context, these findings support the hypothesis that age-related changes contribute to the pathogenesis of neurodegenerative diseases (Zhao and Huai, 2023).

ROS-induced oxidative stress leads to damage to proteins, lipids, and DNA, thereby contributing to brain aging. It is well established that oxidative stress contributes significantly to neuronal dysfunction and the development of neurodegenerative disorders (Singh et al., 2019). In addition to oxidative stress, proinflammatory markers increase with aging, and neuroinflammation is considered an initiating factor in neurodegenerative diseases (Michaud et al., 2013). Protein aggregation (e.g., tau, A*β*, and α-synuclein), a hallmark of neurodegenerative diseases, is also observed in the brains of elderly individuals. A study by Guillozet et al. (2003) demonstrated that both aging and MCI/early Alzheimer’s disease are associated with Aβ aggregation and neurofibrillary tangles. These findings suggest that Alzheimer’s pathology may represent a more advanced form of age-related protein aggregation (Guillozet et al., 2003). APOE allele distribution suggests a possible shared genetic basis between healthy aging and resilience to AD pathology. In this context, APOEε4 is strongly associated with increased AD risk, whereas APOEε2 is linked to healthy aging, longevity, and lower amyloid accumulation (Corder et al., 1994; Grothe et al., 2017). Collectively, these studies indicate that neurodegenerative diseases share common and overlapping molecular and cellular mechanisms with aging.

## 4 Potential therapeutic strategies in aging and neurodegenerative diseases

Aging is a multifactorial process that contributes to the development of many age-related pathologies, particularly neurodegenerative diseases such as Alzheimer’s disease, Parkinson’s disease, Huntington’s disease, and amyotrophic lateral sclerosis. In this context, elucidating the mechanisms of aging is critically important for identifying the fundamental molecular pathways associated with aging and neurodegenerative diseases. Consequently, it becomes possible to develop potential therapeutic strategies targeting processes that trigger disease progression, such as protein misfolding and aggregation, neuroinflammation, proteostasis disruption, and reduced regenerative capacity. Table 2 provides a comprehensive overview of current and experimental therapeutic strategies targeting the common mechanisms of aging and neurodegeneration (Guo et al., 2022) (Table 2).

**Table 2:** Overview of potential therapeutic interventions targeting aging and neurodegeneration related mechanisms, including their proposed mechanisms of action, clinical trial phases, and representative studies.

**Drug/intervention**	**Mechanism**	**Phase of clinical trial**	**Study**
Vaccine (ALZT-OP1a)	Targeting misfolded Aβ oligomers	Phase III	Jeremic et al. (2021)
Monoclonal antibody (Aducanumab)	Targeting Aβ aggregates	FDA approved	Nisticò and Borg (2021)
Vaccine (CAD106)	Inducing antibodies against Aβ	Phase II	Vandenberghe et al. (2016)
BACE1 inhibitor (VERubecestat)	Reduction in Aβ production	Phase II/III (terminated)	Chris Min et al. (2019)
Tau antisense oligonucleotides	Reduction in tau production	Phase I	DeVos et al. (2017)
Tyrosine kinase inhibitor (Nilotinib)	Targeting tau aggregation	Phase II	Nobili et al. (2023)
mTOR inhibitor (Rapamycin)	Enhancing autophagy	Phase II	Subramanian et al. (2022)
GLP-1 receptor agonist (NLY01)	Promoting neurogenesis	Phase II	Park et al. (2021)
Metformin (NCT04098666)	Amyloid β1-40-induced cognitive decline	Active, not recruiting (phase II/III)	Khaleghi-Mehr et al. (2023)
Senolytics (NCT04685590)	Modulation of Alzheimer’s disease progression	Recruiting (phase II)	Gonzales et al. (2022a) and Gonzales et al. (2022b)
Engineered cytokine IL-10 (IL-10 variant/IL-10*)	Modulating neuroinflammation; promoting anti-inflammatory microglial responses, enhancing neurogenesis and cognition	Preclinical (aged mice)	Navarro Negredo et al. (2026)
Tau antisense oligonucleotide (BIIB080)	MAPT-targeting ASO; reduction in tau synthesis and CSF tau, reduction in tau aggregates/NFT burden	Phase II	Edwards et al. (2023) and Mummery et al. (2023)
Tau antisense oligonucleotide (NIO752)	MAPT-targeting ASO; reduction in tau synthesis	Phase Ib/II	Bhandari et al. (2026) and Harris and Hirschfeld (2025)
Flavanone (Liquiritigenin)	Induction of mitochondrial fusion – Protection against Aβ cytotoxicity		Liu et al. (2011) and Jo et al. (2016)
Drug (MitoQ)	Protection from oxidative damage by inhibiting ROS		Hu and Li (2016)
mTOR inhibitor (Rapamycin/rapalogs)	mTOR inhibition; enhancing autophagy and modulating aging-related pathways	Human studies/systematic review	Lee et al. (2024)

Aβ, amyloid-beta; IL-10, interleukin 10; MAPT, microtubule-associated protein tau; ASO, antisense oligonucleotides; NFT, neurofibrillary tangle; GLP-1, Glucagon-like peptide-1 receptor; BACE1, Beta-secretase 1; mTOR, Mechanistic target of rapamycin.

Within this complex mechanistic framework, targeting amyloid-β has emerged as a significant therapeutic strategy in the treatment of Alzheimer’s disease, particularly through active immunization approaches such as ALZT-OP1a and CAD106, as well as monoclonal antibodies including aducanumab (Jeremic et al., 2021; Nisticò and Borg, 2021; Vandenberghe et al., 2016). Meanwhile, small molecule approaches based on BACE1 inhibitors, such as verubecestat, have aimed to reduce Aβ production directly. However, the discontinuation of late-phase clinical trials has underscored the translational challenges associated with these strategies and limited their clinical applicability (Chris Min et al., 2019). Taken together, these findings highlight the therapeutic potential of amyloid-targeting interventions, while also demonstrating that practical challenges continue to limit their clinical success.

In this context, antisense oligonucleotide-based approaches, such as BIIB080 and NIO752, represent promising therapeutic strategies designed to reduce tau production by suppressing MAPT expression. Among them, nilotinib, which targets tau aggregation, is of particular interest. Early clinical findings suggest that these agents successfully engage their targets and may have the potential to modify disease progression (Edwards et al., 2023; Mummery et al., 2023; Bhandari et al., 2026; Harris and Hirschfeld, 2025).

In addition to protein-targeted therapies, some approaches aim to restore homeostatic mechanisms disrupted by aging. Rapamycin enhances autophagy, NLY01 modulates neurogenesis and glial responses, and metformin targets age-related metabolic and cognitive impairments (Subramanian et al., 2022; Park et al., 2021). Furthermore, senolytics and compounds such as MitoQ and liquiritigenin broaden the therapeutic landscape beyond classic amyloid- or tau-focused approaches by targeting cellular senescence and mitochondrial dysfunction (Hu and Li, 2016; Gonzales et al., 2022a; Gonzales et al., 2022b).

Another novel approach focuses on targeting cellular aging and chronic neuroinflammation, two age-related processes that significantly contribute to neurodegenerative pathology. In this context, senolytic therapies may modulate disease progression through the selective elimination of senescent cells, while immunomodulatory strategies, such as IL-10 variants, aim to enhance anti-inflammatory microglial responses, promote neurogenesis, and improve cognitive outcomes by reshaping inflammatory signaling (Gonzales et al., 2022a; Gonzales et al., 2022b; Navarro Negredo et al., 2026). These findings indicate that, in the treatment of neurodegenerative diseases, a more comprehensive and personalized therapeutic framework is emerging that goes beyond approaches targeting a single pathology to address the aging brain microenvironment and interconnected multiple aging mechanisms.

The multifactorial nature of aging and neurodegenerative diseases has led to the development of multi-target therapeutic strategies in recent years. Table 3 summarizes different types of therapeutics including natural compounds, clinical combinations, multi-target directed ligands (MTDLs), and multi-target drugs. These strategies have attracted considerable attention because of targeting key pathological processes simultaneously, including neuroinflammation, oxidative stress, Aβ accumulation, cholinergic dysfunction, glutamatergic dysregulation, and cellular senescence.

**Table 3:** Multi-target therapeutic strategies for aging and neurodegenerative diseases, their mechanisms of action, evidence/status, and supporting studies.

**Drug/intervention**	**Mechanism**	**Evidence/Status**	**Type**	**Study**
Quercetin/Genistein/Epigallocatechin-3-gallate (EGCG)	Suppression inflammatory markers and pro-inflammatory cytokines via NF-κB modulation; reduce neuroinflammation and neuronal degeneration	Preclinical	MTNC	Leyva-López et al. (2016)
Taxifolin	Anti-oxidative, anti-glycation, and anti-Aβ effects; reduces inflammation, oxidative damage, glutamate, and Aβ buildup; improves cerebral blood flow	Preclinical	MTNC	Tanaka et al. (2019), Inoue et al. (2019), and Saito et al. (2017)
Donepezil + Memantine	Simultaneously targets cholinergic dysfunction and glutamatergic dysregulation	Clinical combination; associated with improved 5-year survival in AD patients	CT / MTCC	Yaghmaei et al. (2024)
Donepezil + Memantine + Curcumin	Targeting cholinergic deficits, glutamatergic dysregulation, and neuroinflammation	Investigational multi-target strategy	MTCC	Topal et al. (2022)
ASS234	Inhibition AChE, BuChE, and MAO-A/MAO-B	Experimental multi-target candidate	MTDL	Bautista-Aguilera et al. (2014)
Donecopride	AChE inhibition + 5-HT4 receptor agonism	BBB-penetrant, low-toxicity, precognitive effects *in vivo*	MTDL	Lecoutey et al. (2014)
Rivastigmine–benzimidazole (RIV–BIM) hybrids	Cholinesterase inhibition, metal ion binding, Aβ aggregation inhibition, and antioxidant activity	Experimental multi-target strategy	MTDL	Pathak and Kabra (2024)
Buntanetap (Posiphen/(+)-phenserine lineage)	Translational inhibition of multiple neurotoxic proteins	Clinical	MTD	Fang et al. (2023)
Varoglutamstat	Combined Aβ and inflammation inhibitor	Phase 2A positive; Phase 2B ongoing	MTD	Scheltens et al. (2018)
Dasatinib + quercetin	Targeting senescence-related pathways and inflammation	Phase 1/2 trend only	CT / senolytic	Zhu et al. (2023)
Masitinib	Multi-kinase + FGFR inhibition	Phase 3 cognitive benefit	MTD	Dubois et al. (2023)

MTNC, multi-target natural compound; CT, combination therapy; MTCC, multi-target compound combination; multi-target-directed ligand; MTDL, multi-target-directed ligand; MTD, Multi-Target Drug; AChE, acetylcholinesterase; BuChE, butyrylcholinesterase; MAO, monoamine oxidase; 5-HT4, serotonin type 4; BBB, blood–brain barrier; AD, Alzheimer’s disease; Aβ, amyloid-beta; FGFR, Fibroblast Growth Factor Receptor; NF-Κb, Nuclear Factor kappa B.

In this context, the combination of quercetin, genistein, and EGCG has demonstrated potential to reduce neuroinflammation and neuronal degeneration by suppressing inflammatory markers and pro-inflammatory cytokines through modulation of the NF-κB pathway (Leyva-López et al., 2016). Similarly, taxifolin has promising antioxidant and anti-Aβ effects and may reduce inflammation, oxidative damage, glutamate, and Aβ accumulation (Saito et al., 2017; Tanaka et al., 2019; Inoue et al., 2019).

In addition, the donepezil + memantine combination has improved 5-year survival in AD patients by simultaneously targeting cholinergic and glutamatergic dysfunction (Yaghmaei et al., 2024). Furthermore, the donepezil + memantine + curcumin combination provides a multitarget strategy, targeting a broader pathophysiological framework, including neuroinflammation (Topal et al., 2022).

In parallel, multi-target compounds such as ASS234, donecoprid, and rivastigmine-benzimidazole hybrids have also been developed, combining multiple effects including inhibition of AChE, BuChE, and MAO-A/MAO-B, 5-HT4 receptor agonism, and suppression of Aβ aggregation (Bautista-Aguilera et al., 2014; Lecoutey et al., 2014; Pathak and Kabra, 2024).

Additionally, buntanetap aims to inhibit the translation of multiple neurotoxic proteins, whereas varoglutamstat targets both Aβ pathology and inflammatory pathways (Fang et al., 2023; Scheltens et al., 2018). Moreover, the fact that masitinib demonstrated cognitive benefits in Phase III trials supports the clinical potential of multi-targeted approaches (Dubois et al., 2023). Similarly, the combination of dasatinib and quercetin has also shown promising effects in Phase 1/2 trials by targeting age-related pathways and inflammation (Zhu et al., 2023).

Overall, these findings suggest that strategies targeting multiple interconnected mechanisms may offer a more comprehensive and effective therapeutic framework for aging and neurodegenerative diseases than single-target approaches.

## 5 Conclusion

Brain aging is not only a risk factor for neurodegenerative diseases but also involves a complex network of mechanisms that overlap with disease pathogenesis at the molecular level. Although the mechanisms underlying the transition from healthy aging to neurodegenerative diseases have not yet been fully elucidated, the observation of many characteristic features of the aging brain in neurodegeneration strongly supports the hypothesis of ‘accelerated brain aging’ in these disorders. In this context, the main point is that hallmarks related to aging and neurodegeneration should be considered not as independent processes, but as parts of an integrated network operating through reciprocal interaction.

One of the most significant challenges in this field is the limited availability of high-throughput and physiologically relevant models that reflect both neurodegenerative symptoms and the biology of aging. From this perspective, it is essential that neurodegeneration researchers consider age a fundamental component when developing future models. Moreover, incorporating age into these models may enable a more comprehensive understanding of the multifactorial mechanisms underlying neurodegeneration. Since disease progression is determined by the complex interaction of genetic and environmental factors, a single drug–single target approach is often insufficient. In contrast, multi-target therapeutic approaches and lifestyle-based strategies may offer more effective and sustainable solutions.

Consequently, a more comprehensive characterization of the molecular markers of brain aging and the shared biological pathways underlying neurodegenerative diseases will open new opportunities for more effective and integrated interventions. This approach will not only improve our understanding of disease pathogenesis but also help reduce the growing burden of neurological disorders in an aging population.

## Glossary

AD: Alzheimer’s disease
ALP: Autophagy lysosomal pathway
ALS: Amyotrophic lateral sclerosis
APOE: Apolipoprotein E
APP: Amyloid precursor protein
APP/PS1: Amyloid precursor protein/presenilin-1
ASO: Antisense oligonucleotide
Atg: Autophagy-related protein
ATP: Adenosine triphosphate
Aβ: Amyloid beta
BACE1: Beta-secretase 1
BER: Base excision repair
cGAS–STING: Cyclic GMP-AMP synthase - stimulator of interferon genes
DRP1: Dynamin-1-like protein
ESCRT-III: Endosomal sorting complexes required for transport III
GLP-1: Glucagon-like peptide-1 receptor
GTEx: Genotype-Tissue Expression
HTT: Huntingtin
IL-1β: Interleukin-1 beta
IST1: Increased sodium tolerance-1
LRRK2: Leucine-rich repeat kinase 2
MAPT: Microtubule-associated protein tau
MCI: Mild cognitive impairment
MFN2: Mitofusin 2
mtDNA: Mitochondrial DNA
mTOR: Mechanistic target of rapamycin
NAD^+^: Nicotinamide adenine dinucleotide
OPA1: Mitochondrial dynamin like GTPase
PBL: Peripheral blood leukocytes
PBMCs: Peripheral blood mononuclear cells
PD: Parkinson’s disease
PINK1: PTEN-induced kinase 1
PRKN: Parkin RBR E3 ubiquitin protein ligase
PSEN: Presenilin
ROS: Reactive oxygen species
RTL: Relative telomere length
SNCA: Synuclein alpha
SOD1: Superoxide dismutase 1
SSBs: Single strand DNA breaks
TBI: Traumatic brain injury
TDP-43: TAR DNA-binding protein 43
TER: Telomerase RNA component
TERT: Telomerase reverse transcriptase
TNF-*α*: Tumor necrosis factor-alpha
TREM2: Triggering receptor expressed on myeloid cells 2
UPS: Ubiquitin proteasome system
